# Lumbar Facet Joint Fluid: A Reliable Sign of Lumbar Instability

**DOI:** 10.7759/cureus.39332

**Published:** 2023-05-22

**Authors:** Kulvinder Singh, Trudy Hislop, Ashim Lahiri, Praveen Tekke

**Affiliations:** 1 Department of Radiology, Worcestershire Acute Hospitals NHS Trust, Worcester, GBR; 2 Department of Physiotherapy, Worcestershire Acute Hospitals NHS Trust, Worcester, GBR

**Keywords:** lumbar spine surgery, lumbar spondylolisthesis, spondylolytic listhesis, lumbar facet joint, degenerative lumbar spine

## Abstract

Lumbar degenerative spondylolisthesis (LDS) is a prevalent condition among the elderly population. Magnetic resonance imaging (MRI) is often the first investigative modality if indicated clinically. However, the standard supine position used during an MRI may fail to detect dynamic instability. In such cases, the presence of facet joint fluid is a reliable sign, and further investigation, such as stress radiographs, should be conducted to confirm dynamic instability. Here, we present a typical case demonstrating the importance of this finding. A patient presented with neurological claudication, and an MRI was initially unremarkable except for the presence of lumbar facet joint fluid. This finding prompted us to conduct stress radiographs, which eventually confirmed dynamic instability.

## Introduction

Lumbar degenerative spondylolisthesis (LDS) typically presents with symptoms of spinal stenosis, such as radiculopathy or neurological claudication. It is further categorized as static or dynamic, and it is crucial to identify the dynamic component of LDS before planning for surgery [1,2]. The symptoms of neurogenic claudication in dynamic instability typically worsen while standing, when the lumbar spine is extended and under stress, whereas spinal stenosis is clinically better in the supine position [2]. Similarly, as magnetic resonance imaging (MRI) of the lumbosacral (LS) spine is usually performed in the supine position, it may underestimate the spinal stenosis. The aim of our case report is to emphasize the importance of the presence of lumbar facet joint fluid in an otherwise unremarkable lumbar spine MRI. This sign should prompt further investigation using stress radiographs to accurately diagnose this particular condition.

## Case presentation

Clinical history

A 47-year-old physical instructor presented with pain in the low back and bilateral lateral thighs. The intensity of the pain worsened after walking for five minutes and returned to baseline level with rest in a sitting position. He also experienced pain when turning to the side during sleep. Although there were no significant motor symptoms, he had patchy numbness on both sides of the mid-lateral thigh.

Imaging findings

**Figure 1:**
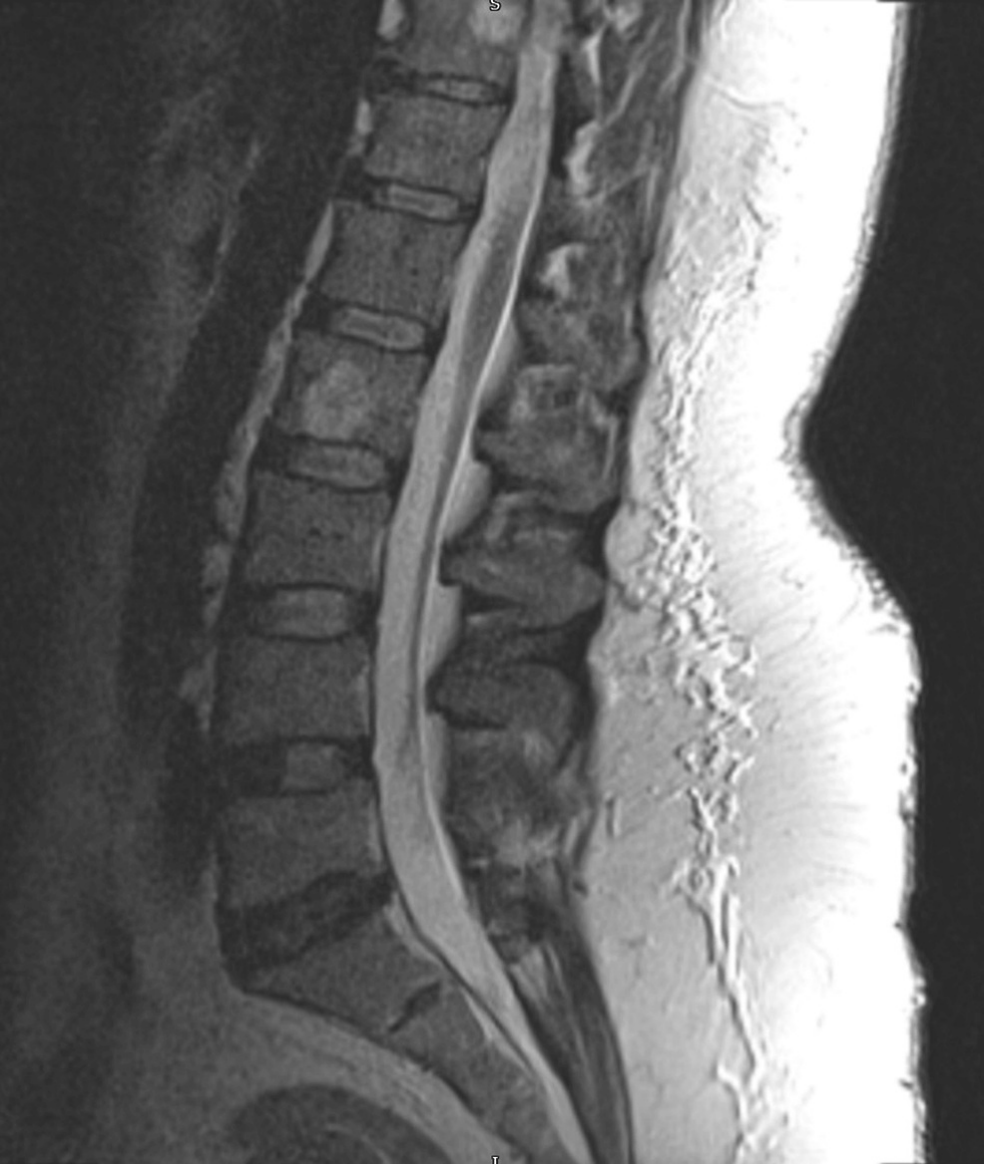
T2W sagittal MRI LS spine showing normal curvature, mild disco-vertebral degeneration, and incidental hemangiomas LS spine, lumbosacral spine; T2W, T2-weighted

**Figure 2:**
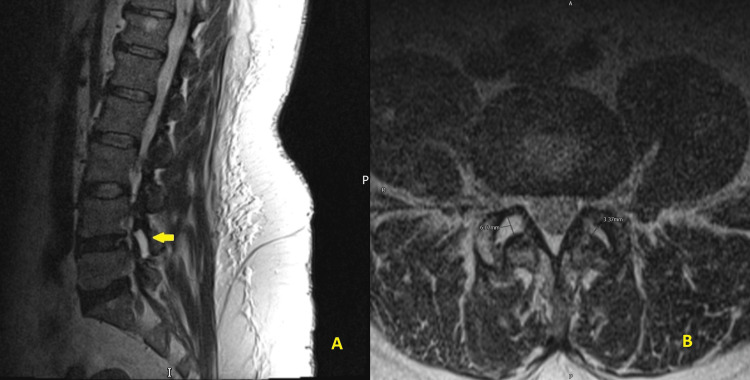
(A) T2W right para sagittal MRI LS spine showing right facet joint fluid (yellow arrow). (B) T2W axial MRI LS spine at L4-L5 intervertebral disc level showing bilateral facet joint fluid with a depth of 6 mm on the right and 3.3 mm on the left side LS spine, lumbosacral spine; T2W, T2-weighted

**Figure 3:**
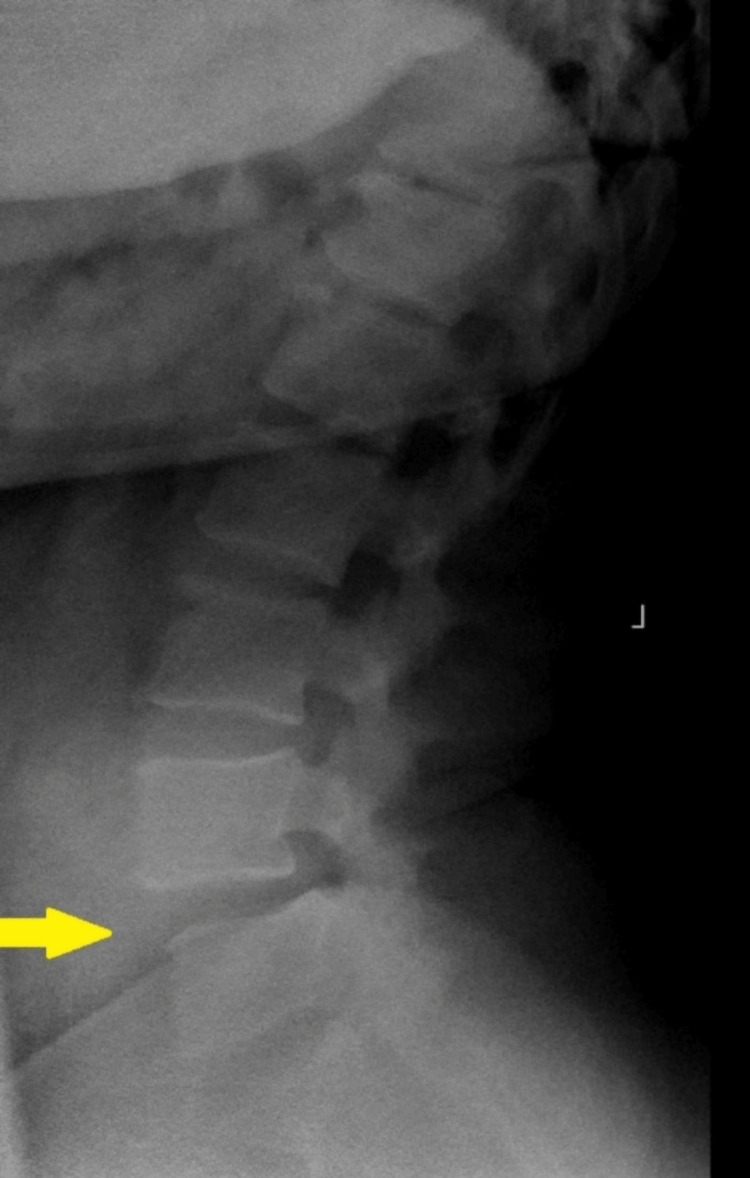
Weight-bearing lateral lumbar radiograph showing grade I anterolisthesis at L4-L5 intervertebral level (yellow arrow)

A supine MRI of the lumbar spine revealed normal lumbar lordosis with mild disco-vertebral degenerative changes (Figure 1). There was asymmetric facet joint effusion at the level of L4-L5, measuring up to 6 mm on the right and 3.4 mm on the left (Figures 2A, 2B). No significant neural compression was observed. A few benign vertebral hemangiomas were incidentally found. Weight-bearing radiographs, both AP and lateral, were taken following the MRI and revealed an exaggerated lumbar lordosis and grade I anterolisthesis of the L4 vertebral body over the L5 vertebral body (Figure 3).

 

 

## Discussion

LDS is a common condition among the elderly population that usually presents with neurogenic claudication and axial back pain [1,3]. Radiologically, there is an abnormal translation of one vertebra over the other in the sagittal plane due to degeneration of the supporting structures of the functional spinal unit. The L4-L5 intervertebral level is most commonly affected, as it is the most mobile segment of the lumbar spine [4].

Although an MRI examination is often the first investigation for the assessment of disco-vertebral degeneration and significant spinal stenosis, conventional supine MRI fails to detect the dynamic component of LDS in more than 1/3 of all cases [5]. Several studies have demonstrated a positive correlation between the presence of facet joint fluid on standard supine MRI LS spines and signs of instability on lateral radiographs [1,4,6].

A synovial joint such as the lumbar facet typically degenerates due to cartilage loss, subchondral ossification, osteophyte formation, and intra-articular fluid accumulation. Studies have shown that facet joint effusion, advanced facet degenerative changes, and synovial cysts are independently associated with degenerative spondylolisthesis [4]. The MRI appearance of lumbar facet joint effusion is seen as a T2-weighted high signal cleft, and the width of effusion corresponds to the amount of joint effusion. Chaput et al. first described facet joint effusion as a radiological predictor of LDS; facet joint distention of 1.5 mm or above was found to be significant [4].

The lumbar spine MRI demonstrating normal curvature and facet joint fluid is considered a reliable sign of dynamic spondylolisthesis with a high positive predictive value (93.22%) [3]. However, additional weight-bearing radiographs are recommended to confirm the diagnosis [3]. Identification of dynamic instability prior to surgical planning is crucial for long-term surgical outcomes since dynamic instability is an independent risk factor for failed laminectomy without fusion [7].

## Conclusions

Lumbar facet joint fluid on standard supine MRI assessment of a clinically suspected LDS is a reliable sign of dynamic instability and should be further followed with stress radiographs for confirmation before surgical planning.

A fluid depth of 1.5 mm or more is a significant measurement.

## References

[1] Aggarwal A, Garg K (2021). Lumbar facet fluid-does it correlate with dynamic instability in degenerative spondylolisthesis? A systematic review and meta-analysis. World Neurosurg.

[2] Even JL, Chen AF, Lee JY (2014). Imaging characteristics of "dynamic" versus "static" spondylolisthesis: analysis using magnetic resonance imaging and flexion/extension films. Spine J.

[3] Cho IY, Park SY, Park JH, Suh SW, Lee SH (2017). MRI findings of lumbar spine instability in degenerative spondylolisthesis. J Orthop Surg (Hong Kong).

[4] Chaput C, Padon D, Rush J, Lenehan E, Rahm M (2007). The significance of increased fluid signal on magnetic resonance imaging in lumbar facets in relationship to degenerative spondylolisthesis. Spine (Phila Pa 1976).

[5] Segebarth B, Kurd MF, Haug PH, Davis R (2015). Routine upright imaging for evaluating degenerative lumbar stenosis: incidence of degenerative spondylolisthesis missed on supine MRI. J Spinal Disord Tech.

[6] Li C, Liu W, Luo W, Zhang H, Zhao J, Gu R (2022). Lumbar facet joint effusion on magnetic resonance imaging: do different joint effusion images have different clinical values?. World Neurosurg.

[7] Blumenthal C, Curran J, Benzel EC (2013). Radiographic predictors of delayed instability following decompression without fusion for degenerative grade I lumbar spondylolisthesis. J Neurosurg Spine.

